# Antibiotic binding of STY3178, a yfdX protein from *Salmonella* Typhi

**DOI:** 10.1038/srep21305

**Published:** 2016-02-19

**Authors:** Paramita Saha, Camelia Manna, Santasabuj Das, Mahua Ghosh

**Affiliations:** 1 Department of Chemical, Biological And Macromolecular Sciences, S. N. Bose National Centre for Basic Sciences, Block JD, Sector III, Salt lake, Kolkata 700098, India; 2 Division of Clinical Medicine, National Institute of Cholera and Enteric Diseases, P-33, C.I.T. Road, Scheme XM Beleghata, Kolkata 700 010, India

## Abstract

The yfdX family proteins are known for long time to occur in various virulent bacteria including their multidrug resistant (MDR) strains, without any direct assigned function for them. However, yfdX protein along with other proteins involved in acid tolerance response is reported to be up regulated by the multidrug response regulatory system in *E. coli*. Hence, molecular and functional characterization of this protein is important for understanding of key cellular processes in bacterial cells. Here we study STY3178, a yfdX protein from a MDR strain of typhoid fever causing *Salmonella* Typhi. Our experimental results indicate that STY3178 is a helical protein existing in a trimeric oligomerization state in solution. We also observe many small antibiotics, like ciprofloxacin, rifampin and ampicillin viably interact with this protein. The dissociation constants from the quenching of steady state fluorescence and isothermal titration calorimetry show that ciprofloxacin binding is stronger than rifampin followed by ampicillin.

Bacteria are one of the major sources for human infections. The bacterial proteins leading to virulence are of fundamental significance. Many of these bacterial proteins have no functional annotation and are termed as domains of unknown function (DUF)^1^. Availability of complete genome sequence for many bacterial strains increases the number of identified DUF proteins whose functional characterization remains grossly incomplete.

Among the DUF proteins, the yfdX protein family is a prominent member. These yfdX proteins have orthologues identified in many virulent bacteria, such as *E. coli, S.* Typhi*, S.* Typhimurium*, S.* Paratyphi, *K. pneumoniae, P. ananatis, E. tarda, H. alvei* and *P. shigelloides*^2^,^3^,^4^,^5^,^6^,^7^,^8^,^9^,^10^,^11^. Many of the strains of these virulent bacteria are also multidrug resistant (MDR). yfdX protein is first identified in *E. coli*^12^,^13^ where the expression of multidrug response regulator protein evgA induced the co-expression of yfdX protein in the cytoplasmic fraction. DNA microarray analysis has further shown that expression of a group of proteins, yfdW, yfdU yfdV, yfdE and yfdX are up regulated by evgA. A significant (orders of magnitude) enhancement of the expression for yfdX gene was observed^13^,^14^ compared to other yfdWUVE proteins upon overproduction of evgA in *E. coli* when quantified using real time PCR. Proteins yfdWUVE are primarily involved in acid tolerance response (ATR) activity^15^. However, yfdX proteins till date remain completely uncharacterized to the best of our knowledge. Occurrence of yfdX proteins in disease-causing bacteria and its co-expression along with the multidrug response regulator protein in *E. coli* indicates that this protein probably has functional role in bacteria which is hitherto unknown. Structure of a yfdX protein from *K. pneumoniae* is reported till date in the protein data bank (PDB 3DZA) which is a tetramer containing metal ions in the monomer interfaces. No functional characterization is reported for this protein as well.

STY3178 is a yfdX protein from the MDR strain (CT18) of *Salmonella* Typhi, the etiologic agent of a potentially lethal febrile illness in the humans^3^. Typhoid fever is a major public health threat to the developing countries worldwide and the concern has significantly increased with the prevalence of MDR strains. *S.* Typhi (CT18) is reported to be resistant^16^,^17^,^18^,^19^,^20^ to many antibiotics like ciprofloxacin (Cpx), rifampin (Rfp), ampicillin (Amp) and so on. The homologues of STY3178 are found across almost all the species of *Salmonella* genus. According to the different database predictions STY3178 is predicted either as a putative membrane protein (Topsan^21^) or as a periplasmic protein (Uniprot^22^ and NCBI). The Kegg^23^ and STRING^24^ databases even mention STY3178 as a completely hypothetical protein.

In the present study we characterize STY3178 protein biophysically and show its interaction with different antibiotic molecules. The questions of our interest are the following: (i) Is STY3178 an oligomeric protein in solution given that its orthologue from *K. pneumoniae* has a tetrameric structure as reported? (ii) Is STY3178 capable of binding drugs or antibiotic molecules? In solution we find STY3178 is a well-folded primarily α-helical protein like its orthologue. Dynamic light scattering (DLS), size exclusion chromatography (SEC) and nuclear magnetic resonance (NMR) relaxation measurements indicate that STY3178 is a trimer. We investigate antibiotic interaction with STY3178 for three different antibiotics to which *S.* Typhi (CT18) is resistant. Finally we quantify the respective binding parameters of these antibiotics to protein and find them in the biologically relevant regime. Our data indicate that ciprofloxacin (Cpx) binds to the protein with higher affinity than rifampin (Rfp) and ampicillin (Amp) binding is weakest among the three.

## Results

**Figure 1:**
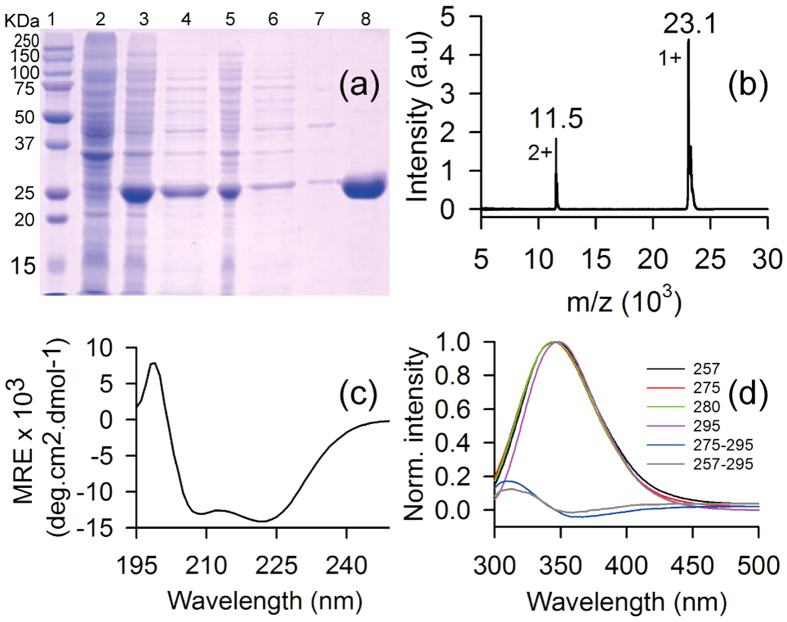
Overexpression, purification, and preliminary characterization of the protein STY3178. (**a**) SDS-PAGE showing the molecular weight marker in lane 1; un-induced cells, lane 2; induced cells, lane 3; crude extract of soluble protein, lane 4; insoluble cell debris, lane 5; flow through from Ni-NTA affinity column, lane 6; wash from Ni-NTA affinity column, lane 7; elute from affinity column, lane 8. (**b**) MALDI-TOF mass spectrum of the purified protein showing peak at 23.1 KDa for the singly charged species. (**c**) Far UV-CD spectrum of the pure protein showing α-helical secondary structure with two characteristic minima around 209 nm and 222 nm. (**d**) Steady state fluorescence emission spectra of the protein for excitation wavelengths 257 (black), 275 nm (red), 280 (green) and 295 nm (magenta). The difference fluorescence spectra (275–295 nm) and (257–295) showing FRET intensity is shown in blue and grey, respectively.

Cloning of our gene of interest (*sty3178)* in prokaryotic expression plasmid pET28a is confirmed by sequencing. Recombinant 6-His-tagged protein (without the N-terminal signal peptide) is expressed successfully in *E. coli* which migrates in SDS-PAGE around ~25 KDa as shown in Fig. 1a (lane 3). High purity of protein is obtained after single step purification as judged from the SDS-PAGE and Coomassie staining (Fig. 1a, lane 8). The calculated molecular weight of the construct is ~23.11 KDa whereas the purified protein migrate around 25 KDa in SDS-PAGE (Fig. 1a, lane 8). We perform mass spectrometry of the purified protein to confirm the actual molecular mass using MALDI-TOF. Figure 1b shows the mass spectrum of the purified protein where the m/z ratio indicates the molecular mass ~23.1 KDa for STY3178. The other peak at 11.5 KDa is assigned for the doubly charged species of the same protein. STY3178 is predicted by Topsan^21^ database as a putative membrane protein, however, we find it in the soluble fraction as observed in the SDS-PAGE (Fig. 1a, lanes 4 and 8). This observation is in complete agreement with the earlier study from *E. coli*^12^ where the orthologues yfdX protein is expressed in the cytoplasm.

The far UV-CD spectrum of purified STY3178 confirms the presence of a well folded protein in solution (Fig. 1c). The spectrum reveals typical characteristic of an α-helix containing protein with two minima at 209 nm and 222 nm, respectively. The estimated^25^,^26^ helix content of the protein using the ellipticity value at 222 nm is ~44%. The ellipticity of the entire complete CD spectrum is further used in Dichroweb server^27^,^28^,^29^ following the K2D^30^ method, we find ~50% helix and ~20% β-sheet content for the protein.

The steady state fluorescence emission spectrum of STY3178 at 20 °C indicates the emission peak position commensurate to Trp emission for any of the following excitation wavelengths 257 nm (black), 275 nm (red), 280 nm (green) or 295 nm (magenta) as shown in Fig. 1d. There are two Phe and five Tyr residues in the protein, but the fluorescence emission from 257 nm and 275 nm excitations are similar to the reported Trp emissions. This indicates a possibility of FÖrster resonance energy transfer (FRET) between Tyr-Trp and Phe-Trp pairs. Difference spectra of 275–295 nm and 257–295 nm excitation wavelengths confirm the signature of FRET between Tyr-Trp (Fig. 1d, blue) and Phe-Trp (Fig. 1d, grey) pairs, respectively. The intensity of FRET is lower for excitation 257–295 compared to 275–295 excitation. At 257 nm excitation all the three aromatic residues could get excited. Phe has a lower quantum yield compared to Tyr or Trp. In addition, Phe emits around 280 nm which is the absorption wavelength for Tyr and Trp resulting in lower intensity of FRET between Phe and Trp (Fig. 1d, grey).

### State of aggregation of the protein

**Figure 2:**
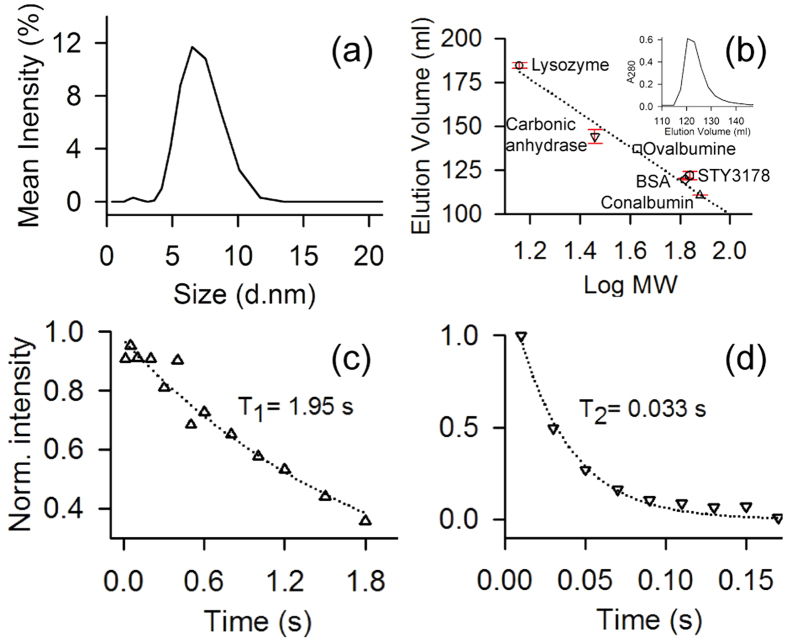
Aggregation state and NMR relaxation measurement for protein STY3178. (**a**) DLS spectrum of the protein showing maximum scattering intensity around a hydrodynamic diameter of 6.5 nm. (**b**) The elution volume versus logarithm of molecular weight of protein in the superdex75 column showing data for lysozyme (14.4 KDa, circle); carbonic anhydrase (29 KDa, inverted triangle); ovalbumin (43 KDa, square); BSA (66 KDa, diamond); STY3178 (hexagon) and Conalbumin (75 KDa, triangle). The error bars are the standard errors estimated using the average elution volume for the repeated experiments. The inset shows the corresponding size exclusion chromatogram for STY3178. (**c**) and (**d**) Show T_1_ and T_2_ relaxation values acquired for the uniformly ^15^N-labelled STY3178 protein, respectively. The delay times used for T_1_ measurements are 0.01, 0.05, 0.1, 0.2, 0.3, 0.4, 0.5, 0.6, 0.8, 1.0, 1.2, 1.5, 1.8 s and for T_2_ measurements are 0.01, 0.03, 0.05, 0.07, 0.09, 0.11, 0.13, 0.15, 0.17 s. The corresponding T_1_ and T_2_ values are extracted by fitting the single exponential decay equation.

Figure 2a shows the average hydrodynamic diameter of the protein in solution ~6.5 nm as observed using dynamic light scattering (DLS). The measured hydrodynamic size is much higher compared to a 23 KDa protein as per the relationship between hydrodynamic size and molecular weight (MW) of different standard globular proteins^31^. For instance, proteins like soyabean trypsine inhibitor (20 KDa) and carbonic anhydrase (29 KDa) have hydrodynamic radius ~2.4 nm and that of ovalbumin (45 KDa) is 2.8 nm as reported earlier^32^,^33^. Thus, a hydrodynamic radius of 3.25 nm for STY3178 would correspond to MW higher than 50 KDa.

The DLS data clearly indicates a size anomaly for the protein in solution considering it as a monomer. To address this anomaly we perform size exclusion chromatography for STY3178 using Superdex75 column (GE healthcare). The elution profile of STY3178 from the column (inset shown in Fig. 2b) is compared with many standard proteins in the molecular weight range of 14 KDa to 75 KDa (Fig. 2b). We find that all these proteins elute from the column at similar volumes repeatedly as indicated by the small error bars in Fig. 2b. STY3178 elutes in the molecular weight regime similar to 66 KDa. This indicates that STY3178 forms a trimer in solution.

We calculate the rotational correlation time (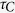) using Stokes-Einstein-Debye (SED) equation 
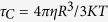
(where 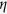 and 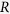 are the viscosity and hydrodynamic radius, respectively) for our measured hydrodynamic radius. Using this relationship we obtain 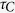 value ~30.5 ns. This 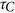 is again much higher compared to protein of similar molecular weight like α-chymotrypsin (25 KDa) where the reported 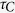 is about 13.4 ns^34^.

We estimate 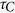 of STY3178 using NMR spectroscopy by acquiring one dimensional (1D) ^1^H-^15^N heteronuclear longitudinal (T_1_) and transverse (T_2_) relaxation data. Since the T_1_ relaxation times are typically longer than T_2_ relaxation times we perform the T_1_ measurement for three different d_1_ delays and average the normalized peak intensities over these measurements. Figure 2c,d show the average peak intensities in 8.5–10.5 ppm region of the spectra plotted against the corresponding delay times in the T_1_ and T_2_ experiments, respectively. The T_1_ and T_2_ values are extracted from the exponential fit of the decays of the integrated peak intensity. We find T_1_ and T_2_ values ~1.95 s and ~0.033 s respectively. The rotational correlation time 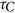 calculated using the experimentally observed T_1_ and T_2_ (as described in methods) is ~24.7 ns. This estimated rotational correlation time is in reasonable agreement with the calculated 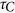 from hydrodynamic radius using the SED equation. Thus all our experimental results indicate a trimeric state of oligomerization for the protein in solution.

### Antibiotic interaction of STY3178

**Figure 3:**
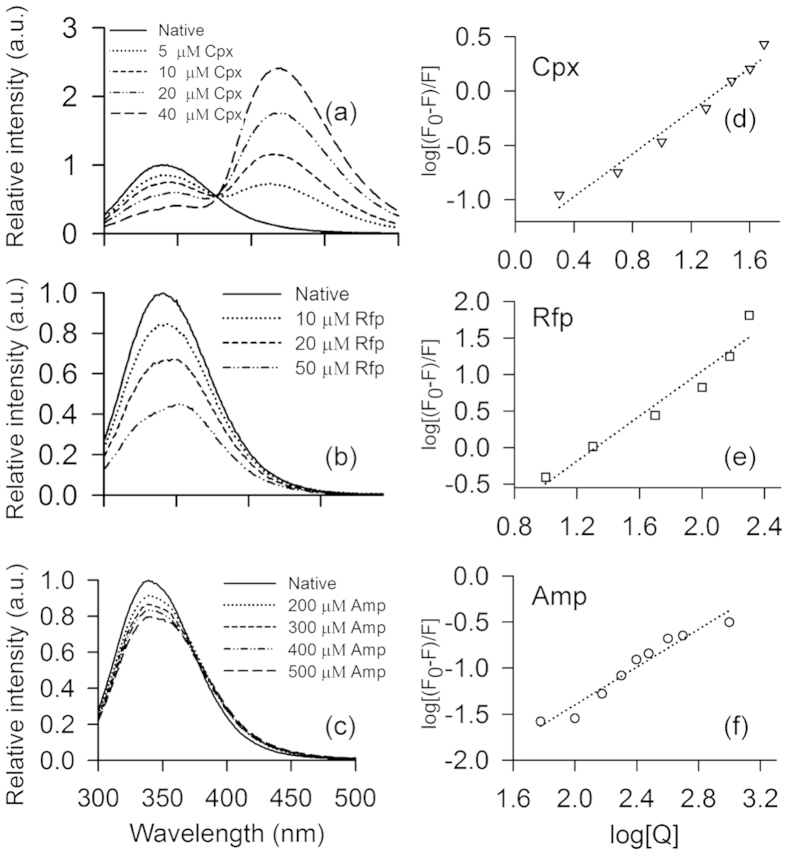
Interaction of STY3178 with antibiotics from fluorescence. (**a–c**) Show the fluorescence emission spectra of STY3178 in presence of different concentrations of ciprofloxacin (Cpx), rifampin (Rfp) and ampicillin (Amp), respectively, for excitation wavelength of 280 nm. (**d–f**) Show the plot of 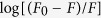 against 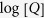 for Cpx (inverted triangle), Rfp (square) and Amp (circle), respectively.

We probe small antibiotic interactions for STY3178 with three different antibiotics, namely, ciprofloxacin (Cpx), rifampin (Rfp) and ampicillin (Amp) using steady state fluorescence spectroscopy, isothermal titration calorimetry (ITC) and circular dichroism. The CT18 strain^3^,^5^ of *S*. Typhi is reported to be resistant to these antibiotics^16^,^17^,^18^,^19^,^20^. We observe fluorescence emission of the protein in presence of antibiotics is quenched considerably compared to the native spectra. A shift in emission peak position for Cpx and Rfp binding (Fig. 3a,b) is observed which is negligible for Amp binding (Fig. 3c). The amount of quenching is, however, different for different antibiotics (Fig.3a–c). We monitor the change in fluorescence intensity with increasing concentration of the antibiotics (Q) for 280 nm excitation wavelength. The fluorescence quenching constant *K*_SV_ is measured using Stern-Volmer equation^35^,^36^,^37^ as described in methods. The value of quenching constant (Table 1) is found different for the three antibiotics. We then measure the binding constant (*K*) for each antibiotic using the modified form of Stern-Volmer equation^35^,^36^,^37^ (see methods). Figure 3d–f show the plots of 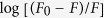 versus log[*Q*] for the three antibiotics Cpx, Rfp and Amp, respectively. The intercept of the fitted curve provides the value for *K* and the slopes provide the information on the number of binding sites (n) as summarised in Table 1. Fluorescence data indicate Cpx and Amp bind to a single site (n≈1). However, Rfp has a possibility of binding in more than one site with n (≈1.5) exceeding unity. The dissociation constant (*K*_d_) is calculated from the reciprocal of *K* (Table 1). We estimate the ΔG for each of these protein-antibiotic interactions from the *K* values and summarise them in Table 1.

**Figure 4:**
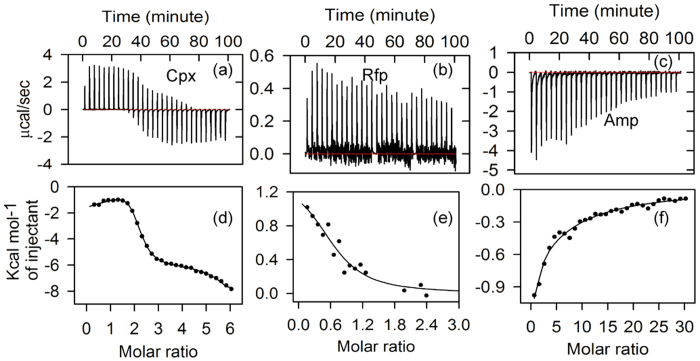
STY3178 interaction with antibiotics from ITC. The thermogram of (**a**) ciprofloxacin (Cpx) bound STY3178, (**b**) rifampin (Rfp) bound and (**c**) Ampicillin (Amp) bound protein. Fitted isotherms are shown for interaction of (**d**) Cpx, (**e**) Rfp and (**f**) Amp with STY3178.

We verify these antibiotic interactions using ITC. The thermograms for Cpx, Rfp and Amp recorded at 298 K are shown in Fig. 4a–c and the corresponding binding isotherms in Fig. 4d–f, respectively. We fit the isotherms in a sequential binding model for Cpx and Amp whereas Rfp data is fitted to a single site model. The binding parameters, including *K*, *K*_d_, enthalpy changes (∆H), ∆G and entropy changes (T∆S) are detailed in Table 2. In the sequential binding model fits, we have considered only the stronger binding parameters. The ITC data indicate binding of Cpx, Rfp and Amp with STY3178 where the dissociation constants belong to the micro molar (μM) range. We observe that Cpx binds stronger than Rfp and Amp. ∆G of Cpx binding is more favourable followed by Rfp and Amp. This trend of binding constants and the ∆G from ITC are similar to those from steady state fluorescence measurements, although the ITC data show much stronger binding.

**Figure 5:**
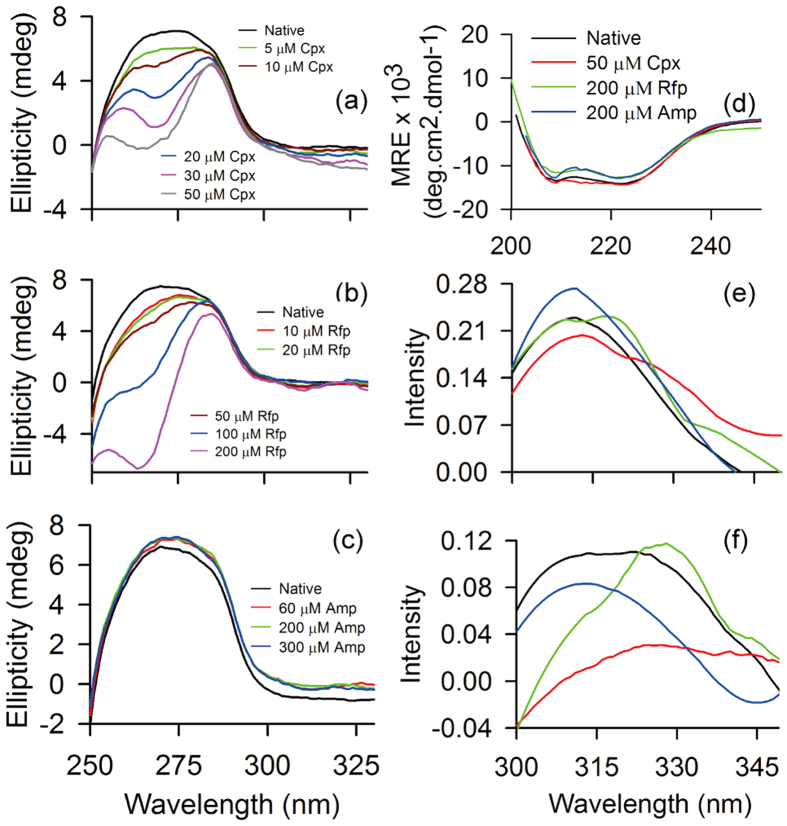
Antibiotic free and bound STY3178 CD spectra and fluorescence difference spectra. (**a–c**) Show the near UV-CD spectra in the range 250–330 nm. The different concentrations of (**a**) ciprofloxacin used are 5 μM (green), 10 μM (brown), 20 μM (blue), 30 μM (magenta) and 50 μM (grey); (**b**) rifampin are 10 μM (red), 20 μM (green), 50 μM (brown), 100 μM (blue), 200 μM (magenta) and (**c**) ampicillin are 60 μM (red), 200 μM (green) and 300 μM (blue). (**d**) Shows the far UV-CD (200 nm- 250 nm) spectra in antibiotic bound and free state. The difference fluorescence emission spectra are shown for (**e**) 275–295 nm and (f) 257–295 nm. The native protein is shown in black, ciprofloxacin (50 μM) bound protein in red, rifampin (200 μM) bound in green and ampicillin (200 μM) bound form in blue in panels (**d–f**).

We probe the structural changes induced in the protein in presence of antibiotics using CD. The near UV-CD spectra of the protein in antibiotic free and bound states are shown in Fig. 5a–c. The native protein structure shows a broad peak around 250–280 nm. This broad peak indicates the involvement of aromatic residues in the tertiary structure of the protein^38^. We observe changes in this broad peak structure in presence of antibiotics. However, the extent of changes is different for different antibiotics and sensitive to their concentrations. For instance, in presence of ciprofloxacin with increasing concentration, two peaks start appearing near ~260 nm and ~280 nm as shown in Fig. 5a. These peaks are similar to Phe and Tyr fine structures as reported in literature^26^,^38^. This indicates that ciprofloxacin interaction with the protein occurs near Phe and Tyr residues. Perturbation of aromatic residues upon addition of rifampin to the protein shows a similar trend like ciprofloxacin binding. The ellipticity values are different for binding with different concentration of ciprofloxacin (Fig. 5a) and rifampin (Fig. 5b) with the peak positions remaining similar. On the other hand, ampicillin binding to the protein (Fig. 5c) shows negligible change compared to the native spectrum.

The far UV-CD spectra in presence of all the three antibiotics are very similar to the native protein structure (Fig. 5d). A slight decrease in ellipticity is observed for rifampin and ampicillin bound protein whereas no substantial change is observed upon ciprofloxacin binding. The percentage of helix content for the native as well as antibiotic bound proteins are tabulated in Table 3 where we find only 3% decrease in helix content for rifampin and ampicillin bound structures when compared to the native. Thus the secondary structural elements remain largely unaffected by the antibiotics.

We further estimate the τ_c_ of the protein in presence of antibiotics to probe any change in the oligomeric state. Table 3 summarises the T_1_, T_2_ and τ_c_ values for the native and antibiotic bound protein. We find no noticeable change in the τ_c_ of the protein in bound state for all the antibiotics. This indicates that the aggregation state of the protein remains same upon binding these small ligands. This is further confirmed from the hydrodynamic radius (R_H_) of the molecule which also remains unchanged for the antibiotic bound protein when measured using DLS as given in Table 3.

## Discussion

We find STY3178 is a well folded and predominantly α-helical protein containing some β-sheet elements. This observation is similar to the secondary structural elements of the orthologues protein structure 3DZA available in the PDB. Our experiments also suggest that STY3178 is an oligomer in solution similar to 3DZA. However, STY3178 is a trimer, whereas the 3DZA structure is as a tetramer. We rule out any possibility of stable tetrameric aggregation for STY3178 based on our experimental observations. STY3178 tetramer would be of MW 92 KDa, had it been a stable tetramer. When we compare the proteins of MW higher than 90 KDa we find the reported hydrodynamic radius for them to be larger than 3.25 nm (Supplementary Table S1). In the elution volume versus log MW plot of SEC data, STY3178 would be a mismatch point from the standard protein line not following the slope if the protein is a tetramer. These observations indicate that the yfdX proteins seem to have a propensity for oligomeric state formation but the degree of oligomerization varies within the family. This is a feature observed for other small oligomeric proteins like family of small heat shock proteins^39^.

Our experimental data demonstrate that different small antibiotics are capable of binding to STY3178. Both steady state fluorescence and ITC results show binding preference towards Cpx, followed by Rfp and Amp. The *K*_d_ values for Cpx binding measured from ITC are an order of magnitude stronger than that measured from fluorescence. Similarly, Amp binding is captured well from ITC, whereas fluorescence data indicate much weaker binding. However, for Rfp binding, the *K*_d_ value is similar as obtained from both the measurements. The stoichiometries of binding from ITC and fluorescence results are not the same. These discrepancies may be due to the fact that fluorescence quenching depends on the binding of the antibiotics in the vicinity of the fluorophores, while ITC is independent of this. The binding modes for all these three antibiotics are different as suggested by different fitting protocols required for the isotherms.

These antibiotics neither perturb the secondary structure as observed from far UV-CD nor affect the oligomeric state of the protein as detected in DLS and NMR relaxation measurements. However, they show tertiary structural rearrangement as observed in the near UV-CD (Fig. 5a–c). The probable residues involved in the interaction are the aromatic residues of the protein. We study the near UV-CD of the isolated aromatic residues in presence of antibiotic molecules. All the three isolated amino acids Phe, Tyr and Trp in presence of Cpx show change in spectra (supplementary Fig. S1a). On the other hand, in presence of Rfp, the changes are small for isolated Phe but isolated Tyr and Trp show pronounced changes (supplementary Fig. S1b) qualitatively similar to that observed for protein. When we compare the nature of perturbation in presence of Amp (supplementary Fig. S1c), very little changes are seen for isolated Phe and Trp but the change in isolated Tyr is again qualitatively similar to the nature of change observed in protein (Fig. 5c).

The native protein fluorescence shows signature of FRET between the aromatic residues (Fig. 1d). In presence of antibiotics, this FRET signature changes (Fig. 5e,f). In Fig. 5e we find an enhancement of FRET upon Amp binding whereas decrease in FRET intensity for Cpx binding. FRET intensity does not change in Rfp bound protein but the difference spectrum shows appearance of another peak different from the native peak ~318 nm having same intensity. In the difference spectra of 257–295 nm shown in Fig. 5f, we observe a decrease in intensity of FRET upon Amp binding and a qualitative change of the overall spectrum in presence of Rfp showing a peak ~330 nm. The FRET signature of 257–295 nm is significantly quenched in presence of Cpx. These observations indicate that the aromatic residues involved in FRET are also associated with antibiotic binding.

Interestingly, we find that similar binding site interactions containing aromatic amino acids are observed in proteins associated with multidrug efflux process. For instance, in BmrR^40^ bound to kanamycin or tetracycline, the antibiotic is stacked near Tyr and Phe residues. Similarly, Phe is found in the vicinity of Rfp in the antibiotic bound structure of AcrB^41^. Amp bound OmpF^42^ has both Phe and Tyr in the near vicinity of the antibiotic. This observation is also seen for other small molecule binding like rhodamine 6G in RamR^43^ where Phe participates in the interaction.

The binding parameters, *K* and *K*_d_ for nearly 100 different protein-antibiotic binding and protein-small molecule interactions^43^,^44^,^45^,^46^,^47^,^48^,^49^ are tabulated in Supplementary Table S2. We observe *K*_d_ values lie in the range 1–100 μM for more than ~80% cases. The next ~15% belong to the range 100–200 μM and less than 5% in the range 200–300 μM. Our data from ITC fall in the lowest *K*_d_ range. Thus, STY3178 shows antibiotic binding capability in the biologically relevant regime.

The blast^50^ search of STY3178 indicates that this yfdX protein is highly conserved among all *Salmonella* species with 92% or higher sequence identity. STY3178 also shares a minimum of 40% sequence similarity with other reported yfdX proteins from various bacteria. Sequence alignment of yfdX proteins from the MDR strains of several bacteria^2^,^3^,^4^,^5^,^6^,^7^,^8^,^9^,^10^,^11^ are shown in supplementary Fig. S2. Such high sequence similarity from different organisms indicates that they might have similar structural fold. However, the order of oligomerization for different yfdX proteins can differ depending on the primary sequence and the size of the protein. The other notable feature that transpires from the sequence alignment is that several locations have conserved Tyr and Phe residues. For instance, we find in 3DZA structure one Phe and two consecutive Tyr residues are in close proximity and these residues are conserved as well. This indicates that 3DZA could be involved in antibiotic interaction like STY3178. Among the yfdX family protein these proximal Phe and consecutive Tyr residues of 3DZA are conserved as well indicating possibility of similar antibiotic interaction as observed for STY3178.

In conclusion, we have characterized the yfdX protein STY3178 from the MDR strain of *S.* Typhi and identified its antibiotic binding ability for the first time to the best of our knowledge. The oligomeric state of the protein in solution is revealed from the biophysical characterization using CD, fluorescence and NMR studies. Our study reveals that yfdX protein, even though not functionally characterized, may not be completely non-functional which in turn opens up further possible studies for this family of proteins. Such studies could be immensely helpful to understand their involvement in pathogenic activity of virulent bacteria.

## Material And Methods

### Cloning

The gene of interest (*sty3178*) with 573 base pair encoding the desired protein is amplified by polymerase chain reaction (PCR) from *S*. Typhi gemonic DNA. The forward and reverse primers used during this amplification are 5′-CATATGGCCGCAACAAACATGACTG-3′ and 5′- CTCGAGGATATTAATGCGCGGCGTCGTG -3′ (Integrated DNA Technologies), respectively. The primers contain the restriction sites for the enzymes NdeI (CATATG) and XhoI (CTCGAG). The amplified PCR product is then inserted into TA vector using T4 DNA ligase and transformed into Top10 *E.coli* cells (Novagen). The transformed bacteria with the desired gene are confirmed by screening the blue/white colonies followed by colony PCR. The plasmid prepared from the transformants is then digested and the insert is purified and sub-cloned into the pET28a expression vector (Novagen). The sub-cloning is confirmed using T7 sequencing primers specific for pET28a expression system. The plasmid containing the desired gene is transformed into *E.coli* BL21(DE3) strain (Novagen).

### Overexpression

Transformed cells containing the plasmid pET28a with *sty3178* gene are grown in 5 ml Luria-Bertani (LB) medium overnight at 37 °C with constant shaking at 250 rpm in a shaker (Innova 42 New Brunswick Scientific). 1% of starter culture is used to inoculate 1 litre of fresh LB and the bacteria are grown till optical density (OD_600_) reaches 0.9. Overexpression is induced by 0.2 mM isopropyl-β-D-thiogalactoside for 4hrs. The cells are then harvested by centrifugation (Eppendorf) at 5000 g for 10 minutes at 4 °C.

### Purification

Harvested cells are resuspended in lysis buffer containing 50 mM potassium phosphate (pH 7), 250 mM sodium chloride (NaCl) and 1 mM phenylmethanesulfonyl fluoride (PMSF) and disrupted by sonication (Sartorius LABSONIC) at 30% amplitude and 0.7 cycle in ice-bath. Cell lysate is centrifuged at 14000 g for 10 minutes at 4 °C and the expressed protein is obtained in the supernatant.

Supernatant containing the expressed protein is purified using Nickel-Nitrilotriacetic acid beads (Qiagen), pre-previously equilibrated with lysis buffer. Beads are washed with four column volumes of buffer containing 50 mM potassium phosphate (pH 7), 250 mM NaCl and 1 mM PMSF and 30 mM imidazole to remove non-specific binding. The recombinant protein is eluted with buffer containing 50 mM potassium phosphate (pH 7), 250 mM NaCl and 1 mM PMSF and 250 mM imidazole.

Protein concentration ~6.2 μM is used in 12% Sodium dodecyl sulphate polyacrylamide gel electrophoresis (SDS-PAGE) to check the purity after affinity chromatography. Imidazole is removed from the sample by buffer exchange using a spin concentrator (10 KDa cut-off, Amicon) and lysis buffer. Concentration of pure protein is determined using Beer-Lambert law and absorbance 280 nm (BioSpectrometer, Eppendorf). Extinction coefficient (ε_280_) from Protparam^51^ tool (Expasy server) obtained for the construct sequence is 18450 M^−1^cm^−1^.

### Mass Analysis

We perform mass spectrometry using MALDI-TOF Bruker Ultraflextreme spectrometer to determine the mass of purified protein. Protein is mixed in 1:1 ratio with sinapinic acid which is dissolved in a mixture of acetonitrile and trifluoroacetic acid (1:1).

### Size Exclusion Chromatography

The affinity chromatography purified protein is subjected to a size exclusion column (2.5 × 45 cm) packed with superdex75 (GE Healthcare) and equilibrated with lysis buffer. Protein concentration used for size exclusion chromatography is ~500 μM. Fractions of purified protein are eluted at a flow rate of 1 ml/min using a peristaltic pump (GE Healthcare). Absorbance at 280 nm wavelength is measured for all the fractions to identify the pure protein. Calibration of the column is performed using standard proteins namely, Lysozyme (14.4 KDa), Carbonic anhydrase (29 KDa), Ovalbumin (43 KDa), BSA (66 KDa) and Conalbumin (75 KDa). The error bars for standard proteins and STY3178 are estimated as standard errors from three sets of repeat experiments.

### Circular Dichroism (CD)

CD measurements are performed in Jasco J-815 CD spectrometer at 20 °C using a quartz cuvette. In the absence and presence of antibiotics, far UV (200–250 nm) and near UV (250–330 nm) protein CD spectra are collected using 1 mm and 10 mm path-length cells, respectively. Concentration of protein used for far UV-CD is 10 μM and near UV-CD is 30 μM. Concentration of antibiotics used are: i) ciprofloxacin 5, 10, 20, 30 and 50 μM; ii) rifampin 10, 20, 50, 100 and 200 μM; iii) ampicillin 60, 200 and 300 μM. Isolated amino acid CD in presence of antibiotics is performed with 400 μM Tyr, 1000 μM Phe and 400 μM Trp. All measurements reported are an average of three scans and buffer subtracted.

Helical content of the protein is calculated using the following relation^25^,^26^ 
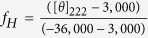
, where 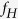
and 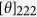 are fractional helicity and mean residue ellipticity at 222 nm.

### Dynamic Light Scattering (DLS)

The measurement is carried out in Nano-S Malvern instrument at 20 °C. Sample concentration of 10 μM is subjected to laser scattering of wavelength 632.8 nm where the measuring angle 173°. The hydrodynamic size of the protein-antibiotic complex is measured in presence of 50 μM ciprofloxacin, 200 μM rifampin and 200 μM ampicillin. Each measurement is obtained as a mean of five successive counts. All the samples prior to each measurement are passed through 0.22 μm syringe filter (Millipore).

### Steady State Fluorescence Spectroscopy

The spectra are recorded using Jobin Yvon Horiba Fluorolog with a slit width of 2 nm. Sample concentration of 10 μM is excited at wavelengths 257, 275, 280 and 295 nm. The final spectrum for each excitation wavelength is obtained after subtracting the lysis buffer spectrum. In presence of increasing concentration of antibiotics (ciprofloxacin, rifampin and ampicillin), the experiment is repeated in a similar way. Each data set represented is an averaged over two sets of measurements.

The fluorescence quenching data upon antibiotic binding to protein is analysed using Stern-Volmer equation^35^,^36^,^37^, 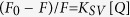 where F_0_ and F, respectively, are the fluorescence intensities in absence and presence of the antibiotic at concentration Q and K_SV_ Stern-Volmer quenching constant. Using the modified form of Stern-Volmer equation^35^,^36^,^37^, 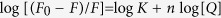, value of binding constant (*K*) and the number of binding sites (n) are estimated along with dissociation constant (*K*_d_) and ∆G using standard protocol^37^.

### Isothermal Titration Calorimetry (ITC)

ITC measurements are performed for STY3178 binding to ciprofloxacin, rifampin and ampicillin using MicroCal iTC200 calorimeter (GE healthcare). Pure protein is dialyzed against buffer containing 30 mM phosphate (pH7), 150 mM NaCl and 1 mM PMSF. Protein concentration used in the cell for these experiments is 300 μM. Antibiotics concentrations loaded in the syringe are 20 mM ciprofloxacin, 10 mM rifampin and 100 mM ampicillin. All titrations including protein- antibiotics and buffer- antibiotics are performed in dialysis buffer. Temperature and reference power used in ITC are 298 K and 10 μcal/s, respectively. A total of 30 injections with an initial delay of 60 sec for each antibiotic titration are performed with constant stirring at 50 rpm. First injection of 0.4 μl over a time period of 0.8 sec is followed by 29 injections of 0.6 μl each for 1.2 sec spaced by 200 sec between each injection. Integrated data after subtracting the heat of dilution for respective antibiotics are plotted using MicroCal origin. Rifampin data is fitted to single site binding model and ciprofloxacin and ampicillin data are fitted to sequential binding models to estimate *K*, enthalpy (∆H) and entropy (∆S). *K*_d_ is calculated from the reciprocal of *K* and change in free energy (∆G) is estimated using Gibbs equation, 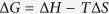.

### NMR experiments

STY3178 protein is uniformly ^15^N-labeled using M9 minimal media supplemented with ^15^NH_4_Cl as a source of nitrogen. It is extracted and purified following the same protocol mentioned above in the purification section.

### Relaxation measurements

The one dimensional (1D) ^1^H-^15^N heteronuclear longitudinal (T_1_) and transverse (T_2_) relaxation experiments are performed at 30 °C using 600 MHz Varian spectrometer equipped with triple resonance probe. The final ^15^N-labeled protein concentration used for the measurement is 355 μM in 30 mM phosphate buffer (pH 7), 150 mM NaCl and 10% D_2_O. For T_1_ measurement, d_1_ time delays of 8, 9 and 12 s are used and that for T_2_ measurement is 4 s. The free induction decay (FID) for T_1_ is collected for the delay points 0.01, 0.05, 0.1, 0.2, 0.3, 0.4, 0.5, 0.6, 0.8, 1.0, 1.2, 1.5 and 1.8 s. Similarly, delays used for T_2_ are 0.01, 0.03, 0.05, 0.07, 0.09, 0.11, 0.13, 0.15 and 0.17 s. All the FIDs are acquired for 256 scans. The data are processed using VnmrJ to obtain the intensity for the range 8.5–10.5 ppm for each set of delay. The intensities for all the d_1_ time delays for both T_1_ and T_2_ data are first normalised for each individual data set and then averaged for all the data sets acquired at each delay point. The plot of intensity versus time is fitted to a single exponential function using SigmaPlot and T_1_ and T_2_ are calculated from the slope of the fitted curve. The total rotational correlation time (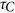) is calculated using the equation^52^,^53^ 
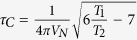
 where *V_N_* is nitrogen (^15^N) resonance frequency in Hertz.

The 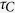 estimations for the antibiotic bound protein are done in the same way as the native protein by measuring the T_1_ and T_2_ using NMR experiments for each bound case.

## Additional Information

**How to cite this article**: Saha, P. *et al.* Antibiotic binding of STY3178, a yfdX protein from *Salmonella* Typhi. *Sci. Rep.* **6**, 21305; doi: 10.1038/srep21305 (2016).

## Supplementary Material

Supplementary Information 1

## Figures and Tables

**Table 1:** STY3178-antibiotic interaction data from steady state fluorescence measurement.

Antibiotic bound	*K*_SV_ (μM^−1^)	*K* (μM^−1^)	*K*_d_ (μM)	n	∆G (KJ mol^−1^)
Cpx	0.05 ( ± 0.002)	0.04 ( ± 0.0023)	25.0 ( ± 1.2)	0.99	−26.5
Rfp	0.26 ( ± 0.034)	0.01 ( ± 0.004)	100.0 ( ± 20.5)	1.54	−22.8
Amp	0.0003( ± 0.00002)	0.0004( ± 0.00004)	2500.0( ± 370.5)	1.03	−14.6

**Table 2:** STY3178-antibiotic binding parameters from ITC.

Antibiotic bound	*K*(μM^−1^)	*K*_d_ (μM)	∆H (KJ mol^−1^)	T∆S (KJ mol^−1^)	∆G (KJ mol^−1^)
Cpx	*K*_1_ = 0.58 ( ± 0.04)	*K*_d1_ = 1.72	∆H_1_ = −6.49 ( ± 0.55)	T∆S_1_ = 26.53	∆G_1_ = −33.02
	*K*_2_ = 0.423 ( ± 0.0079)	*K*_d2_ = 2.36	∆H_2_ = −4.03 ( ± 1.13)	T∆S_2_ = 28.16	∆G_2_ = −32.19
Rfp	*K*_1_ = 0.0151 ( ± 0.00911)	*K*_d1_ = 66.23	∆H_1_ = 6.00 ( ± 1.62)	T∆S_1_ = 29.91	∆G_1_ = −23.91
Amp	*K*_1_ = 0.00744 ( ± 0.0037)	*K*_d1_ = 134.41	∆H_1_ = −6.54 ( ± 1.07)	T∆S_1_ = 15.65	∆G_1_ = −22.19

**Table 3:** Percentage of helix, longitudinal relaxation time (T_1_), transverse relaxation time (T_2_), calculated rotational correlation time (τ_c_) and hydrodynamic radius (R_H_) of native and antibiotic bound STY3178.

State	Helix (%)	T_1_ (s)	T_2_ (s)	τ_c_(ns)	R_H_(nm)
Native protein	43.9	1.95	0.033	24.7	3.25
Protein bound with Cpx	44.5	1.98	0.036	23.8	3.25
Protein bound with Rfp	40.1	2.11	0.033	25.9	3.25
Protein bound with Amp	40.6	1.92	0.034	24.2	3.25
