# Supplementary material for: Antibiotic binding of STY3178, a yfdX protein from Salmonella Typhi
Source: Sci Rep. 2016 Feb 19;6:21305. doi: 10.1038/srep21305 (PMC4759549; doi:10.1038/srep21305)
Supplement: Supplementary Information 1 [file srep21305-s1.doc]

Supplementary Information

Antibiotic binding of STY3178, a yfdX protein from

*Salmonella* Typhi

**AUTHORS**

Paramita Saha^1^, Camelia Manna^1^, Santasabuj Das^2^, and Mahua Ghosh^1*^

^1^Department of Chemical, Biological And Macromolecular Sciences, S. N. Bose National Centre for Basic Sciences, Sector III, Block JD, Salt lake, Kolkata 700098, India

^2^Division of Clinical Medicine, National Institute of Cholera and Enteric Diseases, P-33, C.I.T. Road, Scheme XM Beleghata, Kolkata 700 010, India

**Corresponding Author** Email: *[mahuaghosh@bose.res.in](mailto:mahuaghosh@bose.res.in), *[mahua.ghosh@gmail.com](mailto:mahua.ghosh@gmail.com)

**Supplementary Table S1. Hydrodynamic size ^a^ (R_H_) of various proteins from different molecular weight regimes**

| Protein | Molecuar weight (KDa) | R_H_ (nm) |
| --- | --- | --- |
| Myoglobin | 19.5 | 2.12 |
| Chymotrypsinogen | 25.0 | 2.4 |
| Trypsin inhibitor | 28.0 | 2.47 |
| Carbonic anhydrase | 29.0 | 2.6 |
| Haemoglobin | 65.0 | 3.30 |
| Bovine serum albumin (BSA) | 67.0 | 3.55 |
| Alcohol dehydrogenase | 80.0 | 3.7 |
| Transferrin | 87.1 | 4.0 |
| Amyloglucosidase | 99.0 | 3.9 |
| Hexokinase | 102.0 | 4.3 |

^a^ taken from [www.malvern.co.uk](http://www.malvern.co.uk/)

**Supplementary Table S2.** Antibiotic or small molecule binding parameters for proteins as reported in literature

| S.No. | Protein | Antibiotic | *K*_d_ (μM) | *K* (μM^-1^) | Reference |
| --- | --- | --- | --- | --- | --- |
| 1 | Hemocyanin | Ciprofloxacin |  | 0.05329 | [^1^](#_ENREF_1) |
| 2 | HSA | Oxaliplatin |  | 0.00372 | [^2^](#_ENREF_2) |
| 3 | NorM | Ciprofloxacin | 121.3±15.7 |  | [^3^](#_ENREF_3) |
| 4 | NorM | Proflavin | 33.6±1.9 |  | [^3^](#_ENREF_3) |
| 5 | NorM | Norfloxacin | 105.6±9.8 |  | [^3^](#_ENREF_3) |
| 6 | YdhE | Ciprofloxacin | 90.9±12.4 |  | [^3^](#_ENREF_3) |
| 7 | YdhE | Norfloxacin | 98.4 ±16.2 |  | [^3^](#_ENREF_3) |
| 8 | YdhE | Proflavin | 22.1 ±0.9 |  | [^3^](#_ENREF_3) |
| 9 | OmpF (in DPhPC) | Ceftazidime |  | 0.00607 | [^4^](#_ENREF_4) |
| 10 | AcrB | Proflavin | 14.5 ± 1.1 |  | [^5^](#_ENREF_5) |
| 11 | AcrB | Ciprofloxacin | 74.1 ± 2.6 |  | [^5^](#_ENREF_5) |
| 12 | HSA | Mitoxantrone |  | 4.9 ± 0.8 X 10^-2^ | [^6^](#_ENREF_6) |
| 13 | HSA | Mitoxantrone |  | 2.9 ± 0.3 X 10^-2^ | [^6^](#_ENREF_6) |
| 14 | HSA | Mitoxantrone |  | 2.1 ± 0.5 | [^6^](#_ENREF_6) |
| 15 | HSA | Maslinic acid (MA) |  | 1.42 ± 0.01 X10^2^ | [^7^](#_ENREF_7) |
| 16 | HSA | Quercetin |  | 2.3 ± 0.6 X 10^-2^ | [^8^](#_ENREF_8) |
| 17 | BSA | Quercetin |  | 4.85 ± 0.15 X10^-1^ | [^8^](#_ENREF_8) |
| 18 | BSA | Streptomycin |  | 6.23 X10^-3^ | [^9^](#_ENREF_9) |
| 19 | BSA | Streptomycin |  | (1.74±0.3)X10^-3^ | [^9^](#_ENREF_9) |
| 20 | HSA | Tetracycline |  | 4.94X10 ^-2^ | [^10^](#_ENREF_10) |
| 21 | BSA | Tetracycline |  | 6.2 X 10 ^-2^ | [^10^](#_ENREF_10) |
| 22 | Cephalosporin-binding protein from  *Citrobacter freundii* | Cephalosporin |  | 0.8-2X10^-3^ | [^11^](#_ENREF_11) |
| 23 | HSA | Ciprofloxacin |  | 1.95 × 10^-3^ | [^12^](#_ENREF_12) |
| 24 | EmrE (in SUV) | Proflavin | 10.76± 2.8 |  | [^13^](#_ENREF_13) |
| 25 | EmrE (in SDS) | Proflavin | 4.56± 0.8 |  | [^13^](#_ENREF_13) |
| 26 | EmrE (in DM) | Proflavin | 5.26 ± 0.9 |  | [^13^](#_ENREF_13) |
| 27 | BSA | doxorubicin |  | 7.8±0.7X10^-3^ | [^14^](#_ENREF_14) |
| 28 | HSA | doxorubicin |  | 1.1±0.3X10^-2^ | [^14^](#_ENREF_14) |
| 29 | BSA | Levofloxacin |  | 8.871 X 10^-2^ | [^15^](#_ENREF_15) |
| 30 | BSA | Sparfloxacin |  | 7.2X 10^-2^ | [^15^](#_ENREF_15) |
| 31 | BSA | Enrofloxacin |  | 1.86 X 10^-2^ | [^15^](#_ENREF_15) |
| 32 | BSA | Cefdinir |  | 14.38 X 10^-2^ | [^15^](#_ENREF_15) |
| 33 | HSA | Cytosine β-D arabinofuranoside |  | 2.4 X 10^-3^ | [^16^](#_ENREF_16) |
| 34 | BSA | vincristine sulphate |  | 3.17 X 10^-2^ | [^17^](#_ENREF_17) |
| 35 | BmrR | puromycin | 17 |  | [^18^](#_ENREF_18) |
| 36 | BmrR | tetracycline | 51 |  | [^18^](#_ENREF_18) |
| 37 | BmrR | kanamycin | 28 |  | [^18^](#_ENREF_18) |
| 38 | BmrR | 4-aminoqualdine | 210 |  | [^18^](#_ENREF_18) |
| 39 | BSA | carbenicillin |  | (4.98 ±0.11)X10 ^-3^ | [^19^](#_ENREF_19) |
| 40 | Intestinal fatty acid-binding proteins  (I-FABP) | R-Ibuprofen | 51± 11 |  | [^20^](#_ENREF_20) |
| 41 | Intestinal fatty acid-binding proteins  (I-FABP) | S-Ibuprofen | 157 ± 33 |  | [^20^](#_ENREF_20) |
| 42 | Intestinal fatty acid-binding proteins  (I-FABP) | Bezafibrate | 26± 4 |  | [^20^](#_ENREF_20) |
| 43 | Intestinal fatty acid-binding proteins  (I-FABP) | Nitrazepam | 1200± 80 |  | [^20^](#_ENREF_20) |
| 44 | OmpF | Nalidixic acid |  | 10.47 X 10^-3^ | [^21^](#_ENREF_21) |
| 45 | OmpF | Ciprofloxacin |  | 23.44 X 10^-3^ | [^21^](#_ENREF_21) |
| 46 | OmpF | Grepafloxacin |  | 37.15 X 10^-3^ | [^21^](#_ENREF_21) |
| 47 | OmpF | Moxifloxacin |  | 44.67 X 10^-3^ | [^21^](#_ENREF_21) |
| 48 | OmpF | Ciprofloxacin |  | 14.79 X 10^-3^ | [^22^](#_ENREF_22) |
| 49 | HSA | Betulinic acid |  | 1.685 ± 0.01 | [^23^](#_ENREF_23) |
| 50 | BSA | 2-hydroxy-N′-  (3-hydroxybenzylidene) benzohydrazide (HHB) |  | 9.13 X 10^-2^ | [^24^](#_ENREF_24) |
| 51 | BSA | warfarin |  | 3.12 X10^-2^ | [^24^](#_ENREF_24) |
| 52 | BSA | Ibuprofen |  | 6.27X10^-2^ | [^24^](#_ENREF_24) |
| 53 | HSA | Scutellarin |  | 8.32 X 10^-2^ | [^25^](#_ENREF_25) |
| 54 | Trypsin | Oxytetracycline (OTC) |  | 0.7303X10^-1^ | [^26^](#_ENREF_26) |
| 55 | PBP2 | Nitrocefin | 192 ± 24 |  | [^27^](#_ENREF_27) |
| 56 | PBP2 | Cefepime | 1618± 145 |  | [^27^](#_ENREF_27) |
| 57 | PBP2 | Ceftazidime | 671 ± 116 |  | [^27^](#_ENREF_27) |
| 58 | PBP2 | Ampicillin | 668 ± 124 |  | [^27^](#_ENREF_27) |
| 59 | PBP2 | Oxacillin | 180 ± 25 |  | [^27^](#_ENREF_27) |
| 60 | PBP2 | Imipenem | 603 ± 93 |  | [^27^](#_ENREF_27) |
| 61 | HSA | catechin |  | 1.21 ± 0.52 X10^-2^ | [^28^](#_ENREF_28) |
| 62 | BSA | olanzapine |  | 10.28 X10^-2^ | [^29^](#_ENREF_29) |
| 63 | HSA | cis-fac-[RuIICl2(DMSO)3(KTZ)] |  | 3.3 X 10^-1^ | [^30^](#_ENREF_30) |
| 64 | HSA | cis-[RuIICl2(bipy)(DM SO)(KTZ)] |  | 3.8 X 10^2^ | [^30^](#_ENREF_30) |
| 65 | HSA | [RuII(η6-p-cymene)Cl2(KTZ)] |  | 1.9 | [^30^](#_ENREF_30) |
| 66 | HSA | [RuII(η6-pcymene)(en)(KTZ)][BF4]2 |  | 1.5 X 10^-1^ | [^30^](#_ENREF_30) |
| 67 | HSA | [RuII(η6-p-cymene)(bipy)(KT Z)][BF4]2 |  | 4.4 X 10^2^ | [^30^](#_ENREF_30) |
| 68 | HSA | [RuII(η6-p-cymene)(acac)(KTZ)][BF4] |  | 7.7 X 10^2^ | [^30^](#_ENREF_30) |
| 69 | Apotransferrin | cis-fac-[RuIICl2(DMSO)3(KTZ)] |  | 4.0 X 10^-2^ | [^30^](#_ENREF_30) |
| 70 | Apotransferrin | cis-[RuIICl2(bipy)(DM SO)(KTZ)] |  | 5.3 X 10^2^ | [^30^](#_ENREF_30) |
| 71 | Apotransferrin | [RuII(η6-p-cymene)Cl2(KTZ)] |  | 3.3 X 10^-2^ | [^30^](#_ENREF_30) |
| 72 | Apotransferrin | [RuII(η6-pcymene)(en)(KTZ)][BF4]2 |  | 1.5 X 10^-2^ | [^30^](#_ENREF_30) |
| 73 | Apotransferrin | [RuII(η6-p-cymene)(bipy)(KT Z)][BF4]2 |  | 5.9 X 10^2^ | [^30^](#_ENREF_30) |
| 74 | Apotransferrin | [RuII(η6-p-cymene)(acac)(KTZ)][BF4] |  | 3.8 X 10^2^ | [^30^](#_ENREF_30) |
| 75 | HSA | Hippuric acid |  | (6.84±0.19 )X 10^-3^ | [^31^](#_ENREF_31) |
| 76 | HSA | Camptothecin (CPT) |  | 2.16± 0.33X10^-3^ | [^32^](#_ENREF_32) |
| 77 | BSA | Ferulic acid |  | 97.87 ± 0.03 X10^-2^ | [^33^](#_ENREF_33) |
| 78 | HSA | virstatin |  | (6.09± 0.87) X10^-1^ | [^34^](#_ENREF_34) |
| 79 | HSA | warfarin |  | (3.18± 0.54) X10^-1^ | [^34^](#_ENREF_34) |
| 80 | EhpR | griseoluteic acid | 244 ± 45 |  | [^35^](#_ENREF_35) |
| 81 | albus G | Cephalothin | 9.5 X10^3^ |  | [^36^](#_ENREF_36) |
| 82 | albus G | Cephalosporin C | 1.6 X10^3^ |  | [^36^](#_ENREF_36) |
| 83 | R61 | Cephaloglycine | 0.4 X10^3^ |  | [^36^](#_ENREF_36) |
| 84 | R61 | Ampicillin | 7.2 X10^3^ |  | [^36^](#_ENREF_36) |
| 85 | R61 | Carbenicillin | 0.11 X10^3^ |  | [^36^](#_ENREF_36) |
| 86 | R61 | Cephalosporin C | >1 X10^3^ |  | [^36^](#_ENREF_36) |
| 87 | R61 | Phenoxymethylpenicillin | >1 X10^3^ |  | [^36^](#_ENREF_36) |
| 88 | R61 | Benzylpenicillin | 13 X10^3^ |  | [^36^](#_ENREF_36) |
| 89 | R39 | Cephalosporin C | 0.19 X10^3^ |  | [^36^](#_ENREF_36) |
| 90 | FmtA | Bocillin | 60 |  | [^37^](#_ENREF_37) |
| 91 | Plasma protein | isometamidium | 2.04 |  | [^38^](#_ENREF_38) |
| 92 | phospholipase A_2_ (PLA_2_) | minocycline (minoTc) | 1.8 X 10^2^ |  | [^39^](#_ENREF_39) |
| 93 | YndB | flavanone | 32 ±3 |  | [^40^](#_ENREF_40) |
| 94 | YndB | flavone | 62± 9 |  | [^40^](#_ENREF_40) |
| 95 | YndB | flavonol | 86 ±16 |  | [^40^](#_ENREF_40) |
| 96 | OprD^+Sias^ | piperacillin | (7.60 ±0.13 ) |  | [^41^](#_ENREF_41) |
| 97 | OprD^+Sias^ | ceftazidime | (9.08±0.93) |  | [^41^](#_ENREF_41) |
| 98 | BSA | olmesartan |  | 0.0912 | [^42^](#_ENREF_42) |
| 99 | BSA | olmesartan medoxomil |  | 0.8658 | [^42^](#_ENREF_42) |
| 100 | PBP 2a | Cefepime | 1620 ± 145 |  | [^43^](#_ENREF_43) |
| 101 | PBP 2a | Ceftazidime | 670 ± 115 |  | [^43^](#_ENREF_43) |
| 102 | PBP 2a | Nitrocefin | 190 ± 25 |  | [^43^](#_ENREF_43) |
| 103 | PBP 2a | ceftaroline | 20 ± 4 |  | [^44^](#_ENREF_44) |
| 104 | CG2496, a functionally uncharacterized protein | methiothepin | 54± 19 |  | [^45^](#_ENREF_45) |
| 105 | BSA | Diclofenac sodium |  | 0.1 | [^46^](#_ENREF_46) |
| 106 | FPPS | Bisphosphonate 1 (pH7.4) |  | 13.1 | [^47^](#_ENREF_47) |
| 107 | FPPS | Bisphosphonate 1 (pH8.5) |  | 2.31 | [^47^](#_ENREF_47) |
| 108 | FPPS | Bisphosphonate 2 (pH7.4) |  | 5.07 | [^47^](#_ENREF_47) |
| 109 | FPPS | Bisphosphonate 2 (pH8.5) |  | 1.43 | [^47^](#_ENREF_47) |
| 110 | FPPS | Bisphosphonate 3 (pH7.4) |  | 20.4 | [^47^](#_ENREF_47) |
| 111 | FPPS | Bisphosphonate 3 (pH8.5) |  | 4.6 | [^47^](#_ENREF_47) |
| 112 | FPPS | Bisphosphonate 4 (pH7.4) |  | 0.313 | [^47^](#_ENREF_47) |
| 113 | FPPS | Bisphosphonate 4 (pH8.5) |  | 0.32 | [^47^](#_ENREF_47) |
| 114 | FPPS | Bisphosphonate 5 (pH7.4) |  | 6.02 | [^47^](#_ENREF_47) |
| 115 | FPPS | Bisphosphonate 5 (pH8.5) |  | 3.44 | [^47^](#_ENREF_47) |
| 116 | FPPS | Bisphosphonate 6 (pH7.4) |  | 3.12 | [^47^](#_ENREF_47) |
| 117 | TtgR | phloretin |  | 2.1±0.4 X10 | [^48^](#_ENREF_48) |

**Supplementary Figure S1.** Near UV-CD spectra of isolated amino acids in presence of various antibiotics. The near UV-CD spectra of Phe (F, solid), Tyr (Y, dash) and Trp (W, dotted) in presence of (a) 50 μM ciprofloxacin (brown), (b) 100 μM rifampin (blue) and (c) 200 μM ampicillin (green).


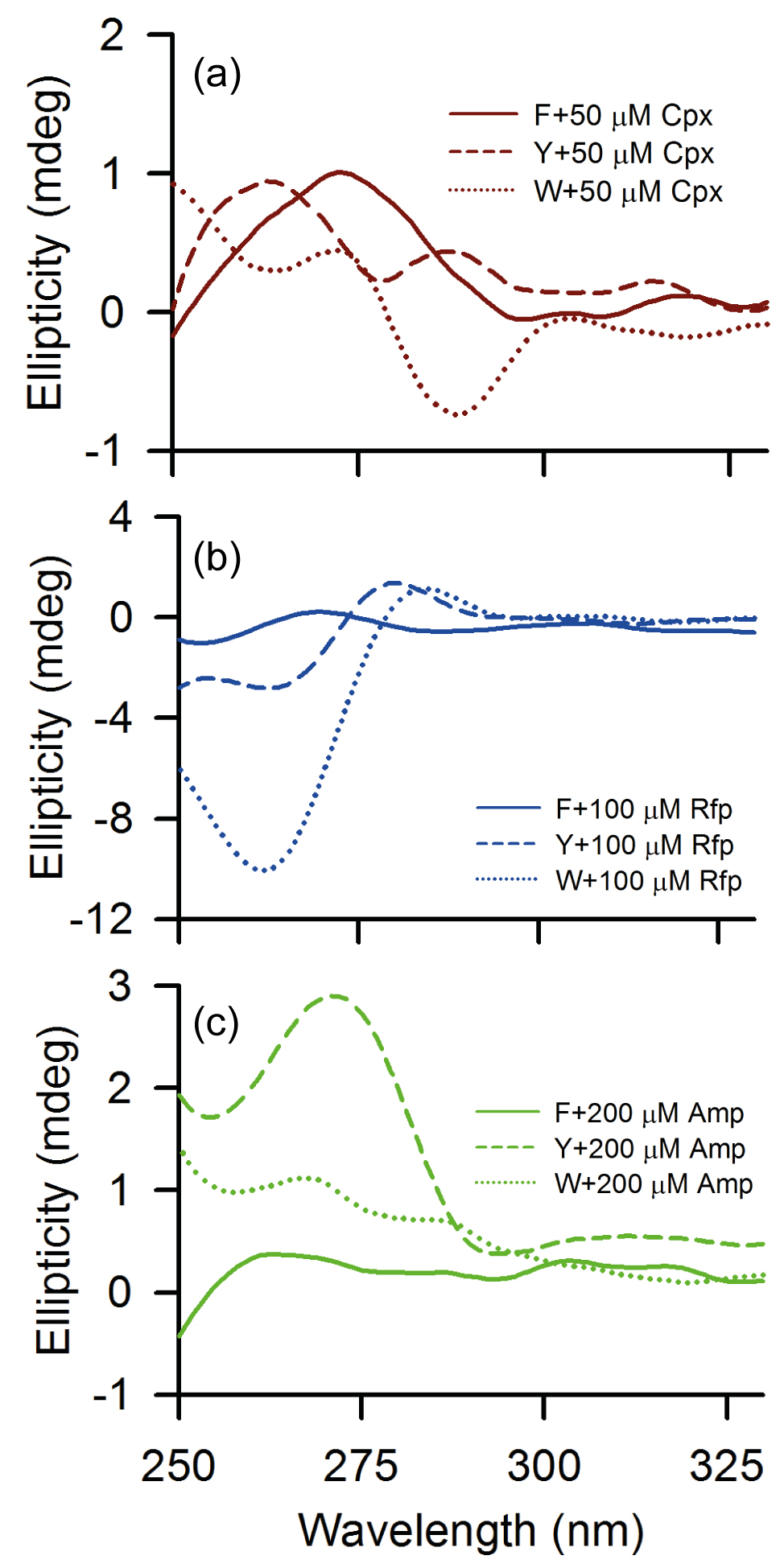


**Supplementary Figure S2.** Sequence alignment of yfdX family proteins. The sequence alignment of STY3178 protein from CT18 strain of *S*. Typhi with the yfdX proteins from other virulent bacteria like *K. pneumoniae, S. enteritidis, S.* Typhimurium*, S.* Paratyphi, *S*. Heidelberg, *S*. Monteviodeo, *P. ananatis, E. tarda, H. alvei, E. coli,*  and *P. Shigelloides* are shown. The secondary structural elements α-helix (cylinder) and β-strands (arrow) are indicated on the top with respect to the crystal structure of yfdX protein (PDB 3DZA) from *K. pneumoniae*. The conserved tyrosine and phenylalanine residues are marked by the rectangular blocks.


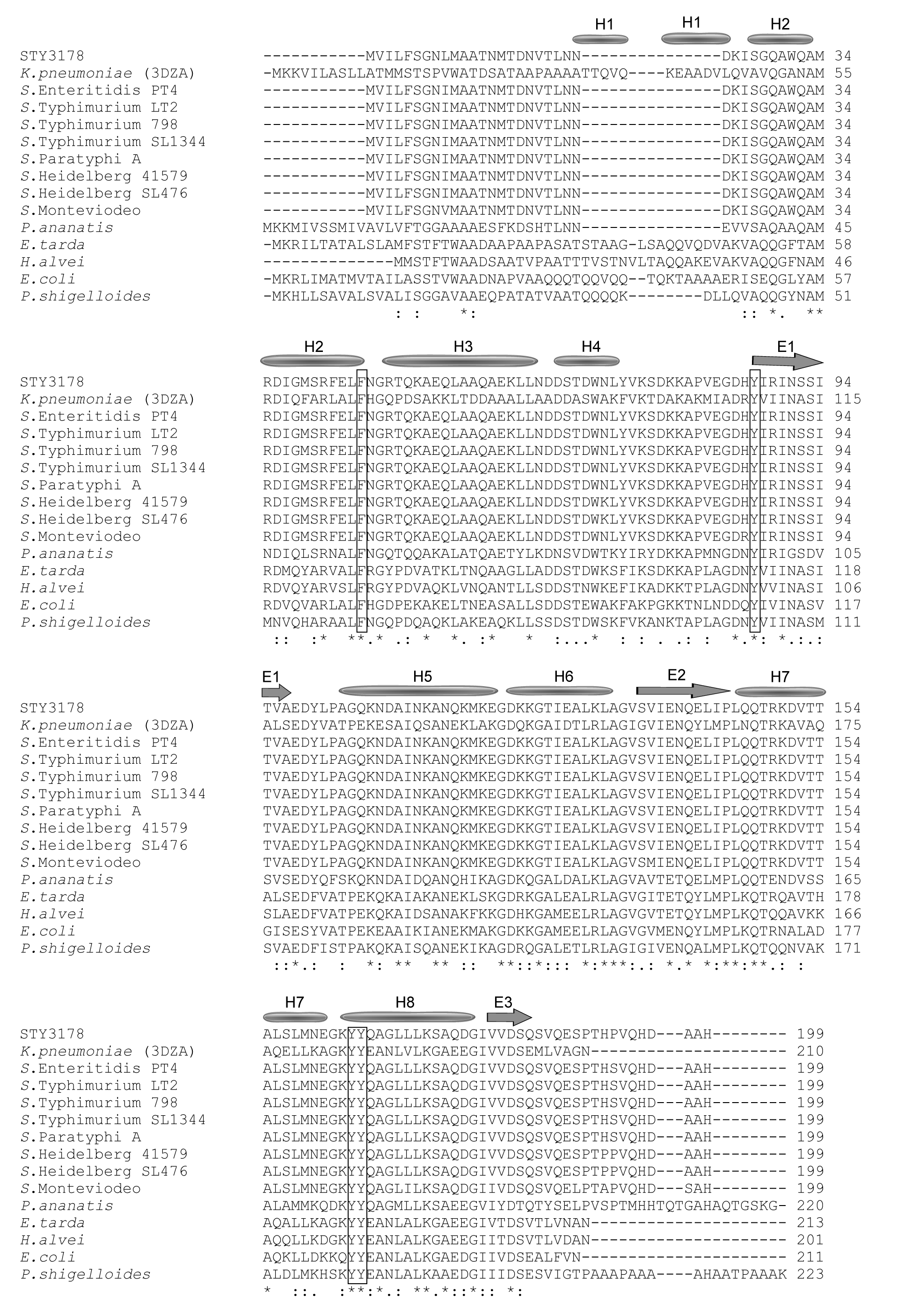


References

1. Zhang HM, Tang BP, Wang YQ, Zhang DZ, Zhang HB. Studies of the interaction between ciprofloxacin and the hemocyanin from chinese mitten crab Eriocheir Japonica Sinensis. *Anal Lett* **44**, 2094-2106 (2011).

2. Yue Y, Chen X, Qin J, Yao X. Spectroscopic investigation on the binding of antineoplastic drug oxaliplatin to human serum albumin and molecular modeling. *Colloids Surf B Biointerfaces* **69**, 51-57 (2009).

3. Long F, Rouquette-Loughlin C, Shafer WM, Yu EW. Functional cloning and characterization of the multidrug efflux pumps NorM from Neisseria gonorrhoeae and YdhE from Escherichia coli. *Antimicrob Agents Chemother* **52**, 3052-3060 (2008).

4. Mihaela Bacalum HW, M. Radu. Interaction between ceftazidime and bacterial porin OmpF analyzed by fluorescence. *Romanian Journal of Biophysics* **19**, 105-116 (2009).

5. Su CC, Yu EW. Ligand-transporter interaction in the AcrB multidrug efflux pump determined by fluorescence polarization assay. *FEBS Lett* **581**, 4972-4976 (2007).

6. Keswani N, Kishore N. Calorimetric and spectroscopic studies on the interaction of anticancer drug mitoxantrone with human serum albumin. *J Chem Thermodyn* **43**, 1406-1413 (2011).

7. Subramanyam R*, et al.* Novel binding studies of human serum albumin with trans-feruloyl maslinic acid. *J Photoch Photobio B* **95**, 81-88 (2009).

8. Mishra B, Barik A, Priyadarsini KI, Mohan H. Fluorescence spectroscopic studies on binding of a flavonoid antioxidant quercetin to serum albumins. *J Chem Sci* **117**, 641-647 (2005).

9. Jha NS, Kishore N. Binding of streptomycin with bovine serum albumin: Energetics and conformational aspects. *Thermochim Acta* **482**, 21-29 (2009).

10. Mukherjee M*, et al.* A Comparative Study of Interaction of Tetracycline with Several Proteins Using Time Resolved Anisotropy, Phosphorescence, Docking and FRET. *Plos One* **8**, (2013).

11. H O. A specific cephalosporin-binding protein of Citobacter Freundii. *Biochim Biophys Acta* **420**, 155–164 (1976).

12. Iranfar H, Rajabi O, Salari R, Chamani J. Probing the interaction of human serum albumin with ciprofloxacin in the presence of silver nanoparticles of three sizes: multispectroscopic and zeta potential investigation. *J Phys Chem B* **116**, 1951-1964 (2012).

13. Sikora CW, Turner RJ. Investigation of ligand binding to the multidrug resistance protein EmrE by isothermal titration calorimetry. *Biophys J* **88**, 475-482 (2005).

14. Agudelo D, Bourassa P, Bruneau J, Berube G, Asselin E, Tajmir-Riahi HA. Probing the binding sites of antibiotic drugs doxorubicin and N-(trifluoroacetyl) doxorubicin with human and bovine serum albumins. *PLoS One* **7**, e43814 (2012).

15. Pooja SNaA. MECHANISM OF INTERACTION OF SOME ANTIMICROBIAL AGENTS WITH BOVINE SERUM ALBUMIN. *Journal of Global Pharma Technology* **2(3)**, 77-86 (2010).

16. Alam P*, et al.* Biophysical and molecular docking insight into the interaction of cytosine beta-D arabinofuranoside with human serum albumin. *J Lumin* **164**, 123-130 (2015).

17. Kamat BP, Seetharamappa J. Mechanism of interaction of vincristine sulphate and rifampicin with bovine serum albumin: A spectroscopic study. *J Chem Sci* **117**, 649-655 (2005).

18. Bachas S, Eginton C, Gunio D, Wade H. Structural contributions to multidrug recognition in the multidrug resistance (MDR) gene regulator, BmrR. *Proc Natl Acad Sci U S A* **108**, 11046-11051 (2011).

19. Thoppil AA, Sharma R, Kishore N. Complexation of beta-lactam antibiotic drug carbenicillin to bovine serum albumin: energetics and conformational studies. *Biopolymers* **89**, 831-840 (2008).

20. Velkov T*, et al.* The interaction of lipophilic drugs with intestinal fatty acid-binding protein. *J Biol Chem* **280**, 17769-17776 (2005).

21. Neves P, Berkane E, Gameiro P, Winterhalter M, de Castro B. Interaction between quinolones antibiotics and bacterial outer membrane porin OmpF. *Biophys Chem* **113**, 123-128 (2005).

22. Fernandes F, Neves P, Gameiro P, Loura LM, Prieto M. Ciprofloxacin interactions with bacterial protein OmpF: modelling of FRET from a multi-tryptophan protein trimer. *Biochim Biophys Acta* **1768**, 2822-2830 (2007).

23. Subramanyam R, Gollapudi A, Bonigala P, Chinnaboina M, Amooru DG. Betulinic acid binding to human serum albumin: A study of protein conformation and binding affinity. *J Photoch Photobio B* **94**, 8-12 (2009).

24. Tong JQ*, et al.* Probing the adverse temperature dependence in the static fluorescence quenching of BSA induced by a novel anticancer hydrazone. *Photoch Photobio Sci* **11**, 1868-1879 (2012).

25. Tian JN, Liu JQ, He WY, Hu ZD, Yao XJ, Chen XG. Probing the binding of scutellarin to human serum albumin by circular dichroism, fluorescence spectroscopy, FTIR, and molecular modeling method. *Biomacromolecules* **5**, 1956-1961 (2004).

26. Chi Z, Liu R, Zhang H. Noncovalent interaction of oxytetracycline with the enzyme trypsin. *Biomacromolecules* **11**, 2454-2459 (2010).

27. Fuda C, Suvorov M, Vakulenko SB, Mobashery S. The basis for resistance to beta-lactam antibiotics by penicillin-binding protein 2a of methicillin-resistant Staphylococcus aureus. *J Biol Chem* **279**, 40802-40806 (2004).

28. Li XR, Wang S. Study on the interaction of (+)-catechin with human serum albumin using isothermal titration calorimetry and spectroscopic techniques. *New J Chem* **39**, 386-395 (2015).

29. Mohammad A Rashid1 SNIR, Tania Sultana, Md. Zamil Sultan and Md. Zakir Sultan. Fluorescence Spectroscopic Study of Interaction between Olanzapine and Bovine Serum Albumin. *Pharmaceutica Analytica Acta* **6**, (2015).

30. Estrada JG, Sanchez-Delgado RA. Spectroscopic Study of the Interactions of Ruthenium-Ketoconazole Complexes of Known Antiparasitic Activity with Human Serum Albumin and Apotransferrin. *Journal of the Mexican Chemical Society* **57**, 169-174 (2013).

31. Zaidi N, Ajmal MR, Rabbani G, Ahmad E, Khan RH. A Comprehensive Insight into Binding of Hippuric Acid to Human Serum Albumin: A Study to Uncover Its Impaired Elimination through Hemodialysis. *Plos One* **8**, (2013).

32. Ishtikhar M*, et al.* Biophysical and molecular docking insight into interaction mechanism and thermal stability of human serum albumin isoforms with a semi-synthetic water-soluble camptothecin analog irinotecan hydrochloride. *J Biomol Struct Dyn*, 1-49 (2015).

33. Ojha H, Mishra K, Hassan MI, Chaudhury NK. Spectroscopic and isothermal titration calorimetry studies of binding interaction of ferulic acid with bovine serum albumin. *Thermochim Acta* **548**, 56-64 (2012).

34. Chatterjee T, Pal A, Dey S, Chatterjee BK, Chakrabarti P. Interaction of virstatin with human serum albumin: spectroscopic analysis and molecular modeling. *PLoS One* **7**, e37468 (2012).

35. Yu S*, et al.* Atomic resolution structure of EhpR: phenazine resistance in Enterobacter agglomerans Eh1087 follows principles of bleomycin/mitomycin C resistance in other bacteria. *BMC Struct Biol* **11**, 33 (2011).

36. Frere JM, Geurts F, Ghuysen JM. The exocellular DD-carboxypeptidase-endopeptidase of Streptomyces albus G. Interaction with beta-lactam antibiotics. *Biochem J* **175**, 801-805 (1978).

37. Fan X, Liu Y, Smith D, Konermann L, Siu KW, Golemi-Kotra D. Diversity of penicillin-binding proteins. Resistance factor FmtA of Staphylococcus aureus. *J Biol Chem* **282**, 35143-35152 (2007).

38. Suprita Sinha SAaTKM. Study of plasma protein binding activity of isometamidium and its impact on anthelmintic activity using trypanosoma induced calf model. *Vet World 6* **7**, 444-448 (2013).

39. Dalm D, Palm GJ, Aleksandrov A, Simonson T, Hinrichs W. Nonantibiotic properties of tetracyclines: structural basis for inhibition of secretory phospholipase A2. *J Mol Biol* **398**, 83-96 (2010).

40. Stark JL*, et al.* Solution structure and function of YndB, an AHSA1 protein from Bacillus subtilis. *Proteins* **78**, 3328-3340 (2010).

41. Khatua B, Van Vleet J, Choudhury BP, Chaudhry R, Mandal C. Sialylation of outer membrane porin protein D: a mechanistic basis of antibiotic uptake in Pseudomonas aeruginosa. *Molecular & cellular proteomics : MCP* **13**, 1412-1428 (2014).

42. SHARMA RN, PANCHOLI, SHYAM S. Protein binding interaction study of olmesartan medoxomil and its metabolite olmesartan by fluorescence spectroscopy. *International Journal of Pharmacy and Pharmaceutical Sciences* **6**, 726-729 (2014).

43. Fuda C*, et al.* Mechanistic basis for the action of new cephalosporin antibiotics effective against methicillin- and vancomycin-resistant Staphylococcus aureus. *J Biol Chem* **281**, 10035-10041 (2006).

44. Fishovitz J*, et al.* Disruption of Allosteric Response as an Unprecedented Mechanism of Resistance to Antibiotics. *Journal of the American Chemical Society* **136**, 9814-9817 (2014).

45. Stark JL, Copeland JC, Eletsky A, Somerville GA, Szyperski T, Powers R. Identification of low-molecular-weight compounds inhibiting growth of corynebacteria: potential lead compounds for antibiotics. *ChemMedChem* **9**, 282-285 (2014).

46. Dutta SK, Basu SK, Sen KK. Binding of diclofenac sodium with bovine serum albumin at different temperatures, pH and ionic strengths. *Indian J Exp Biol* **44**, 123-127 (2006).

47. Yin F, Cao R, Goddard A, Zhang Y, Oldfield E. Enthalpy versus entropy-driven binding of bisphosphonates to farnesyl diphosphate synthase. *J Am Chem Soc* **128**, 3524-3525 (2006).

48. Alguel Y*, et al.* Crystal structures of multidrug binding protein TtgR in complex with antibiotics and plant antimicrobials. *J Mol Biol* **369**, 829-840 (2007).
